# Gait Variability and Multiple Sclerosis

**DOI:** 10.1155/2013/645197

**Published:** 2013-03-03

**Authors:** Michael J. Socie, Jacob J. Sosnoff

**Affiliations:** ^1^Department of Mechanical Science and Engineering, University of Illinois at Urbana-Champaign, Urbana, IL 61801, USA; ^2^Department of Kinesiology and Community Health, University of Illinois at Urbana-Champaign, 301 Freer Hall, 906 South Goodwin Avenue, Urbana, IL 61801, USA

## Abstract

Gait variability, that is, fluctuations in movement during walking, is an indicator of walking function and has been associated with various adverse outcomes such as falls. In this paper, current research concerning gait variability in persons with multiple sclerosis (MS) is discussed. It is well established that persons with MS have greater gait variability compared to age and gender matched controls without MS. The reasons for the increase in gait variability are not completely understood. Evidence indicates that disability level, assistive device use, attentional requirement, and fatigue are related to gait variability in persons with MS. Future research should address the time-evolving structure (i.e., temporal characteristics) of gait variability, the clinical importance of gait variability, and underlying mechanisms that drive gait variability in individuals with MS.

## 1. Gait Variability

 When one moves repeatedly, there are slight alterations in each individual movement. Traditionally, this variability in movement was viewed as random noise-providing minimal important information [1, 2]. With the introduction of chaos and complexity theory into the life sciences in the 1990s, this negative view of variability was challenged [3–5]. It is now maintained that movement variability is an important clinical phenomenon [6]. 

Gait (i.e., walking) is a complicated process involving coordination of multiple systems within the body (e.g., central nervous, musculoskeletal, and cardiovascular system) [7]. To walk, a person's nervous system must send signals to control a large number of muscles while simultaneously processing sensory information in order to monitor and refine movements, all while maintaining an upright stance [7]. Given the multitude of muscles, and neural processes involved, gait variability likely arises from a combination of factors [8]. 

 There is increasing evidence that gait variability is a quantifiable indicator of walking function [1, 2, 9]. In the past, gait variability was viewed as experimental artifact that should be filtered out to reveal average behavior [10]. However, more recently, evidence has demonstrated meaningful characteristics of gait variability, associated with neural control of walking [1, 2, 9, 10]. Increases in gait variability have been observed in individuals with advanced age [11–14] as well as various neurologically impaired populations, including spinal cord injury [15] and neurological conditions, such as Parkinson's disease [16], dementia [17], and multiple sclerosis [18]. Gait variability has further been associated with motor control function [9, 19], energetic cost of walking [20], and falls [12–14] in various populations.

Currently, there is no gold standard to quantify gait variability, and a number of different analysis techniques have been used [1, 2]. Most commonly, gait variability is quantified using distributional metrics, such as the standard deviation (SD) and coefficient of variation (CV) of kinematic and spatiotemporal gait parameters [1, 2]. It is maintained that SD provides a measure of absolute variability, while CV provides a measure of relative variability [3]. Other nonlinear metrics, such as detrended fluctuation analysis and approximate entropy, have been used to examine the temporal sequential structure of gait variability (i.e., the time-evolving structure of variability) [19]. Since different metrics quantify different aspects of gait variability and operate on different timescales, selection of the appropriate metric(s) for answering a particular research question is critical in investigations of gait variability [2].

## 2. Multiple Sclerosis

 Multiple sclerosis (MS) is a degenerative neurological disease affecting approximately 350,000 people in the USA [21] and upwards of 2 millions worldwide [22]. MS is a progressive disease in which damage to the central nervous system causes widespread dysfunction [23]. Symptoms are highly variable among persons with MS, but typically include sensory, cognitive, and motor impairment [23]. MS results in progressive disability, which is indexed clinically by the Expanded Disability Status Scale (EDSS) [24], a 0–10 scale in which 0.0 represents no impairment due to MS, 4.0 indicates the onset of significant walking impairment, 7.5 indicates wheelchair dependence, and 10.0 represents death due to MS. 

Walking impairment is one of the most commonly reported symptoms of MS and has been reported as one of the most impactful symptoms on the quality of life [25]. Given that walking difficulty is a major symptom of MS, documentation of gait impairment is important for indexing disease progression and rehabilitation in MS. To date, the majority of research focusing on gait dysfunction in persons with MS has focused on subjective clinical assessments [26] and average spatiotemporal parameters. Performance walking tests such as the timed 25-foot walk, 2-minute walk, and 6-minute walk are commonly used to measure gait function in MS [26] and routinely demonstrate that persons with MS walk slower than their peers without MS. Examinations of spatiotemporal parameters have revealed that individuals with MS walk slower, taking shorter, slower steps, and spending more of their gait cycle in double-support than healthy controls and these impairments scale with disability [27–30]. Importantly, deficits in gait have been observed in persons with MS compared to controls even at fixed walking speeds [31, 32].

In addition to average gait parameters and timed walking tests, there has been increasing research to characterize variability of gait parameters and to determine the clinical significance of gait variability in MS. The purpose of this report is to review the current state of the literature concerning gait variability in individuals with multiple sclerosis. Results were collected from a literature search for “gait variability + multiple sclerosis” using PubMed. Fourteen publications were found, and after review, 12 of the articles were considered relevant to the topic. Four other published papers located through the authors' personal knowledge that were not listed on PubMed were included. The following discussion separates observations about gait variability in MS into 2 sections, focusing first on variability during short duration walks (≤10 meters) and second, on gait variability during longer duration walks. Future directions for research on gait variability in MS are also proposed.

## 3. Gait Variability in MS

### 3.1. Gait Variability during Short Walks

 The majority of research concerning gait variability in individuals with MS characterizes variability of gait parameters over relatively short distance walks (≤10 meters). Understanding walking behavior, including gait variability, during shorter walks may be applicable to many activities of daily life such as maneuvering about the home or crossing the street. Another potential explanation for why investigations focus on shorter walks is the limitations of data collection equipment. Pressure sensitive electronic walkways are commonly used to measure gait parameters but are typically less than 10 meters long. Additionally, data collection using optical motion capture for overground walking is limited by the capture size. However, the development and refinement of accelerometer-based mobility monitoring has been implemented successfully in persons with MS [33–36] and may potentially provide means to measure gait variability over larger distances.

One of the first investigations to report on variability of walking function examined variability in the timed 25-foot walk. Importantly, variability in walking performance was viewed as random fluctuations (e.g., noise) that must be filtered to reveal significant changes in walking function [37]. The goal of the investigation (*N* = 133, EDSS ranging from 1.0 to 3.5) was to quantify natural variability in timed 25-foot walk performance in persons with MS over a 1-year period in order to determine a threshold for “meaningful change” in performance. Results demonstrated that timed 25-foot walk performance may naturally vary up to 20% over a 1-year period in persons with MS [37]. Therefore, it is suggested that changes in timed 25-foot walk time of 20% or more indicate meaningful change in walking function. It was also found that those with greater disability had the greater variability in 25-foot walk performance than those with less variability. This observation indicates that variability in performance-based test is related to disability. 

There have been multiple reports documenting increased variability of kinematic and spatiotemporal gait parameters in individuals with MS compared to healthy controls during walks of up to 10 meters [18, 27, 38, 39]. In one relatively small report, individuals with MS (*N* = 20, age = 43 ± 10, median EDSS = 3.0) demonstrated significantly greater standard deviation of hip, knee, and ankle angles (SD_hip_ = 2.0°, SD_knee_ = 2.7°, SD_ank_ = 1.5°) during overground walking than healthy controls (*N* = 8, age = 41 ± 9, SD_hip_ = 1.8°, SD_knee_ = 2.3°, SD_ank_ = 1.4°) [18]. Another investigation reported that 43 individuals with MS with minimal disability (*N* = 43, age = 47 ± 9, median EDSS = 2.0) demonstrated greater coefficients of variation for step time (CV_ST_ = 2.6%) and single-support time (CV_SST_ = 3.2%) than age and gender matched controls without MS (*N* = 43, age = 47 ± 10, CV_ST,SST_ = 1.9%, 2.3%) [27]. Meanwhile, others reported a greater CV of step length in persons with MS with minimal impairment (*N* = 9, age = 42 ± 9, median EDSS = 2.0, CV_SL_ = 1.3%) compared to controls (*N* = 9, age = 42 ± 10, CV_SL_ = 1.1%) [38]. Overall, these studies highlight that persons with MS with minimal disability have elevated gait variability compared to control participants. 

In order to examine whether gait variability is influenced by disability in persons with MS, Socie and colleagues [39] quantified gait variability in 88 persons with MS with a wide range of disability (EDSS range = 2.0–6.5; median = 4.5). Participants walked at a comfortable pace along a 26-foot pressure sensitive walkway that recorded spatiotemporal parameters. Congruent with the field, persons with MS demonstrated greater CV of both step time (CV_ST_ = 4.7%) and step length (CV_SL_ = 5.1%) compared to healthy controls (*N* = 20, age = 51 ± 9, CV_ST,SL_ = 1.8%, 2.0%) [39]. Additionally, a positive correlation between EDSS and CV and SD of step time and step length and a negative correlation between EDSS and CV and SD of step width was observed [39]. That is to say, variability of step length and step time is greater in individuals with MS with greater disability, while variability of step width is smaller in persons with MS with greater disability. Similarly, another study with a smaller sample (*N* = 10) which examined walking on a motorized treadmill demonstrated that individuals with MS with EDSS scores ≥4.0 had greater stride length CV (5.0%) compared to a group of individuals with MS with EDSS scores <4.0 (CV_STL_ = 3.2%) [40].

Together the findings of these studies suggest that increases in gait variability occur early on in the disease process and apparently worsen as disability increases. The association between disability and gait variability has only been examined cross-sectionally, so no causal relation should be assumed. It is interesting to note that none of these investigations found a significant difference in persons with MS and step width variability. The differential effect of MS on gait variability of propulsion metrics (e.g., step length, step time, etc.) and gait variability of stability metrics (e.g., step width) is congruent with the notion that stability gait parameters (e.g., step width) and propulsion gait parameters (e.g., step length, step time) are controlled by separate neural circuits that could be differently influenced by disability [41, 42].

### 3.2. Gait Variability during Longer Duration Walks

While most research in MS has focused on gait variability within walks of up to 10 meters, there are some investigations of gait variability in MS during longer walks (>10 meters). The 6-minute walk test is a common clinical gait test in which gait variability has been characterized in individuals with MS. Preliminary evidence suggests that individuals with MS who use assistive devices (*N* = 9) have greater variability of step time (CV_ST_ = 11.0%) and step length (CV_SL_ = 11.6%) than individuals with MS who walk independently (*N* = 9, CV_ST_ = 2.8%, CV_SL_ = 2.3%) and healthy controls (*N* = 10, CV_ST_ = 2.1%, CV_SL_ = 1.8%) throughout the 6-minute walk test [43]. Furthermore, individuals with MS who used assistive devices experienced significantly greater increases in variability of step time, length, width, and double-support time in the last 2 minutes of the test compared to the first 2 minutes compared with the independently ambulatory MS group and controls. The authors suggest that changes in gait variability could be related to changes in stability and fatigue over the course of the 6-minute walk test [43].

In another study, nonlinear dynamics were used to examine variability structure over the course of 3 minutes of walking in individuals with MS (*N* = 10, age = 35 ± 9, median EDSS = 4.0) and healthy controls (*N* = 10, age = 35 ± 10) [40]. Participants with and without MS walked on a motorized treadmill at a comfortable self-selected speed. The long duration of walking allowed for the examination of the time sequential structure of fluctuations in gait. Individuals with MS demonstrated lower approximate entropy of stride length (ApEn = 0.55) and stride width (ApEn = 0.51) than healthy controls (ApEn = 0.70, 0.68 for stride length, width, resp.). Lower approximate entropy values are suggested to indicate more repeatable fluctuations in MS gait and theorized to result in reduced capacity to overcome perturbations [40]. However, the same data showed no differences in detrended fluctuation analysis, another nonlinear measure of variability, of stride length, and width variability between persons with MS and controls [40]. It is important to note that the use of a treadmill could potentially change walking behavior [44], including gait variability. Future work should examine the structure of gait variability in persons with MS in overground walking. 

## 4. Mechanisms of Gait Variability

 Although it is clear that persons with MS have greater amounts of gait variability in short and long walking tasks, the mechanisms underlying this change in gait are not clear. Based on the notion that the control of gait involves numerous neural processes and coordination of the trunk and limbs, it most likely is not a single individual mechanism, but rather a combination of deficits that contribute to gait variability. A potential factor for the increase in gait variability among persons with MS is an increase in noise within the neuromuscular system. Simulations of walking have demonstrated that an increase in neuromuscular signal noise leads to increased gait variability [45]. Indeed, elevated neuromuscular noise has been previously suggested to contribute to impairment in MS [46]. However, specific evidence linking gait variability to neuromuscular signal activity in MS has not been reported. Additionally, empirical evidence of noise within the neuromuscular system is lacking [47] and has been theoretically challenged [5]. 

Another facet of MS that has been studied in connection to gait variability is fatigue, which is a common symptom of MS [23] and is related to MS gait impairment [48]. However, there is limited evidence of associations between fatigue and gait variability. Two studies report that, despite changes in self-reported fatigue, there were no significant changes in gait variability throughout a 24–48 hour period in persons with MS [18, 49]. Preliminary data from our laboratory demonstrates that step time variability and double support variability increase in the persons with MS who use assistive devices while walking during the course of a 6 MW [43]. It was proposed that this increase in gait variability results from an increase in fatigue. Overall, it appears that fatigue may indeed be associated with gait variability in MS; however, the current evidence does not support this assertion in walks of less than 10 meters.

Muscle strength may be another contributing factor to gait variability in persons with MS. Muscle quality (ratio of muscle strength to lean muscle mass, i.e., functional muscle strength) has been associated with gait variability in healthy older adults [11]. Additionally, decreased muscle strength as well as deficits in balance and proprioception are related to gait variability in elderly individuals [8]. Given that persons with MS have decreased muscle strength, proprioception, and balance and that these characteristics is related to gait impairment [50, 51], these factors may contribute to gait variability in MS. 

 An additional factor that maybe related to elevated gait variability in persons with MS is spasticity. Spasticity, the hyperexaggerated stretch reflex, is very common in persons with MS and has been found to be related to gait dysfunction in persons with MS [52, 53]. Moreover, it is logical to speculate that the presence of spasticity in the lower limbs leads to an alteration in the outcome of the descending motor command driving walking. Regardless of the logic of this speculation, there is no data in support of this proposition.

 It is also possible that ataxic movement disorders that are often characterized by hypermetric and dysmetric movements are associated with gait variability in persons with MS. This is a very logical and seemingly clinically evident association. There is evidence that persons with cerebellar ataxia have greater gait variability than healthy controls and that the amount of gait variability is related to walking speed [54, 55]. However, there is no evidence linking ataxia and gait variability in persons with MS. 

 Lastly, there is some evidence to suggest that attentional resources are related to gait variability in persons with MS. One investigation demonstrated that individuals with MS (*N* = 18, age = 39 ± 8, median EDSS = 2.5) exhibited less variability of swing time (CV_SWT_ = 3.0%) in normal walking than while walking and simultaneously performing a cognitive task involving serial addition (CV_SWT_ = 4.0%) [56]. The additional neurological demand of performing a cognitive task likely decreases the amount of control over gait, leading to increased gait variability. 

## 5. Clinical Significance of Gait Variability

To date the clinical significance of gait variability in persons with MS is unknown. However, gait variability has been found to have clinical import in various other special populations. For instance, gait variability is associated with falls in the elderly [8, 12, 13]. There is limited evidence linking gait variability and falls in MS. One investigation [57] demonstrated that recurrent fallers (i.e., 1^+^ falls/year) with MS exhibit greater variability of spatial footfall placement than nonfallers with MS. Spatial footfall placement variability was quantified in that investigation using a novel method involving fitting footfall patterns with Fourier series [57]. Importantly, traditional gait variability metrics (CV of step length, time, and width) did not distinguish between recurrent and nonfallers with MS. To our knowledge, no other data specifically linking gait variability and falls in MS has been published.

It is also possible that elevated gait variability in persons with MS is related to the amount of energy required to walk. Recently, researchers have shown that experimental increases in gait variability are related to increased energetic cost of walking in healthy young adults [20]. The authors propose that individuals with greater gait variability need to spend energy not only on propelling the center of mass, but also on movements to correct for the erratic foot placement. Although it is well established that that persons with MS do indeed have higher energetic cost of walking [58–60], there is no data that this is associated with gait variability. 

It is logical to speculate that since energetic cost of walking is related to fatigue, gait variability is also related to fatigue. To date evidence in support of any association between gait variability and fatigue is ambiguous. Some reports of gait variability during short walks report minimal association between gait variability and fatigue [18, 49]. However, one study on fatigue in MS (*N* = 14, age = 42 ± 8, median EDSS = 3.5) showed significant associations between gait variability and fatigue in MS during walks to exhaustion [61]. In this investigation, participants with MS walked until they felt complete exhaustion. During walks to exhaustion, gait variability was significantly correlated with the motor sections of the Fatigue Scale for Motor and Cognition (FSMC) [61]. Correlations between gait variability and the cognitive portion of the FSMC were not significant, suggesting that physical fatigue is more closely related to gait variability than cognitive fatigue during walks to exhaustion in MS [61].

## 6. Future Directions

Additional research is still needed to further characterize gait variability in MS. For instance, only one relatively small (*N* = 10 MS) study has analyzed time-evolving structure of gait variability in MS [40]. Nonlinear variability measures yield different information than distributional measures (e.g., coefficient of variation) and are indicative of motor function and health and are believed to be more sensitive to dysfunction than traditional measures [6, 9, 19]. Further investigations are needed to characterize the temporal characteristics of gait variability in MS to supplement the growing body of distributional gait variability data that has been reported. 

As well as expanding the scope of metrics used to analyze gait variability in MS, research is warranted to explore possible associations between gait variability and falls in persons with MS. Gait variability has been associated with falls in other clinical populations such as the elderly [12–14]; however, there is limited evidence linking gait variability and falls in MS [57]. Given that 50% of individuals with MS fall in a given year [62, 63], an association between gait variability and falls in MS is clinically relevant.

The mechanisms underlying elevated gait variability in persons with MS need further investigation. There are other factors that have been associated with gait variability in other populations. Determination of these mechanisms would inform interventions designed to maximize mobility including gait variability. Specifically, gait variability could potentially be altered through therapeutic intervention, although specific observations have not been reported. Interventions have demonstrated the ability to improve physical and cognitive performance, disability level, and quality of life in MS [21]. Future interventions in MS, particularly those targeting mobility and gait, should document gait variability as a unique indicator of gait function. Additionally, by documenting gait variability during therapeutic interventions, researchers may also be able to identify specific mechanisms that modulate gait variability in MS.

## 7. Conclusion

Overall, the existing body of research demonstrates that gait variability is elevated in individuals with MS [18, 27, 38–40] and potentially clinically significant. Additionally, a number of factors have been linked to gait variability in MS, including disability level [39], assistive device use [39, 63], dual-task performance [56], and fatigue [61]. However, further research is needed to more fully characterize and to understand the clinical impact of gait variability in individuals with MS.
